# Atypical diabetes subtypes in Black African populations

**DOI:** 10.1007/s00125-026-06697-3

**Published:** 2026-03-02

**Authors:** Jean Claude Katte, Charlotte Bavuma, Sarah H. Wild, Meredith Hawkins, Nihal Thomas, Eugene Sobngwi, Moffat J. Nyirenda, Davis Kibirige

**Affiliations:** 1 Department of Clinical and Biomedical Sciences, University of Exeter Medical School, Exeter, UK; 2 Department of Non-Communicable Diseases Research, RSD Institute, Yaoundé, Cameroon; 3 School of Medicine and Pharmacy, College of Medicine and Health Sciences, University of Rwanda, Kigali, Rwanda; 4 Department of Medicine, University Teaching Hospital, Kigali, Rwanda; 5 Usher Institute, University of Edinburgh, Edinburgh, UK; 6 Global Diabetes Institute, Diabetes Research Centre, Albert Einstein College of Medicine, Bronx, NY USA; 7 Department of Endocrinology, Diabetes, and Metabolism, Christian Medical College, Vellore, India; 8 Centre for Stem Cell Research, Christian Medical College, Vellore, India; 9 Department of Internal Medicine and Specialties, Faculty of Medicine and Biomedical Sciences, University of Yaoundé 1, Yaoundé, Cameroon; 10 National Obesity Centre and the Endocrinology and Metabolic Diseases Unit, Yaoundé Central Hospital, Yaoundé, Cameroon; 11 Department of Non-Communicable Diseases Epidemiology, Faculty of Epidemiology and Population Health, London School of Hygiene and Tropical Medicine, London, UK; 12 Non-Communicable Diseases Program, Medical Research Council/Uganda Virus Research Institute and London School of Hygiene and Tropical Medicine Uganda Research Unit, Entebbe, Uganda; 13 Department of Medicine, Uganda Martyrs Hospital Lubaga, Kampala, Uganda

**Keywords:** Africa, African populations, Atypical diabetes subtypes, Fibrocalculous pancreatic diabetes, Ketosis-prone diabetes, Malnutrition-related diabetes, Review, Type 2 diabetes in individuals without overweight and obesity

## Abstract

Atypical diabetes subtypes and presentations are disproportionately prevalent in populations of African and Asian ancestry. This review discusses the epidemiology, clinical presentation, aetiopathogenesis and management of four atypical diabetes subtypes commonly reported in Black African populations. These are ketosis-prone diabetes (KPD), fibrocalculous pancreatic diabetes (FCPD), type 2 diabetes in individuals without overweight or obesity, and malnutrition-related diabetes (MRD). The review summarises current insights into these atypical diabetes subtypes in Black African populations and provides practical recommendations to guide their precision diagnosis and management in the African region. These four atypical diabetes subtypes exhibit phenotypic features that diverge from those of classical type 1 and type 2 diabetes. KPD is characterised by unprovoked, transient, index episodes of diabetic ketoacidosis, often in the absence of markers of islet cell autoimmunity, with frequent subsequent insulin independence and diabetes remission. FCPD typically presents in young lean individuals, with a strong male preponderance and with radiological evidence of pancreatic calcifications, reduced beta cell reserve and severe hyperglycaemia without ketosis. Type 2 diabetes in individuals without overweight or obesity is characterised by normal BMI with a trend towards low levels of markers of visceral adiposity, insulin resistance and an exaggerated beta cell secretory dysfunction. MRD is associated with a previous and persistent history of undernutrition, with features of undernutrition such as stunting and BMI <18.5 kg/m^2^, resistance to diabetic ketoacidosis, no evidence of visceral or ectopic adiposity, and severe beta cell secretory dysfunction. The high prevalence and heterogeneous presentation of these atypical forms of diabetes in African populations highlight the urgent need for enhanced collaborative research to better define their epidemiology, improve diagnostic accuracy and develop context-appropriate management strategies tailored to diverse African populations.

**Graphical Abstract:**

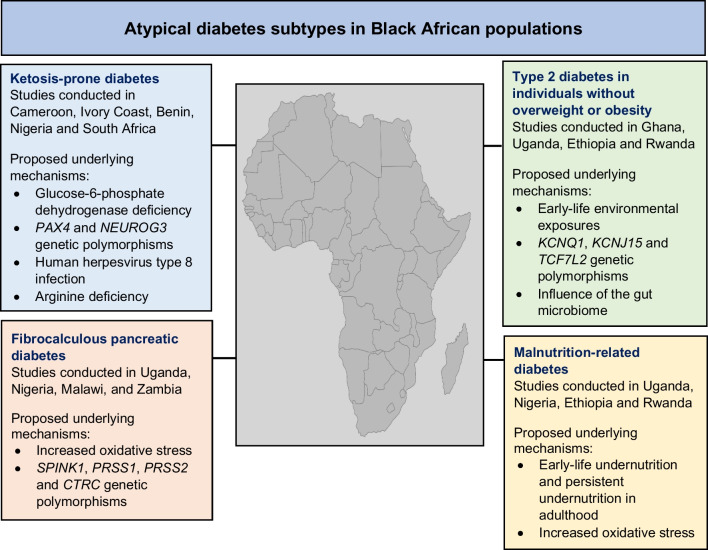

## Introduction

Diabetes is a complex metabolic disorder characterised by marked heterogeneity in clinical presentation, aetiopathogenesis and response to treatment [1]. With advances in phenotyping approaches, several atypical diabetes subtypes have been identified that do not fit the traditional classification of type 1 diabetes and type 2 diabetes [1, 2]. This growing complexity provides significant diagnostic and therapeutic challenges, particularly in low- and middle-income countries (LMICs) where limited access to essential diagnostic tools further complicates clinical decision-making.

This review discusses the published literature on four atypical diabetes subtypes described in Black African populations: ketosis-prone diabetes (KPD), fibrocalculous pancreatic diabetes (FCPD), type 2 diabetes in individuals without overweight or obesity (BMI of <25 kg/m^2^ or <23 kg/m^2^ in some African populations) and malnutrition-related diabetes (MRD). The salient phenotypic characteristics distinguishing these four atypical diabetes subtypes are summarised in Table 1. Other forms of syndromic and non-syndromic genetic atypical diabetes subtypes, such as latent autoimmune diabetes in adults, MODY and severe insulin resistance syndromes, are outside the scope of this review.

**Table 1:** Comparison of the salient phenotypic characteristics of the four atypical diabetes subtypes

Phenotypic characteristic	Atypical diabetes subtypes			
	KPD	FCPD	T2D-NOW	MRD
Age at onset	Third to fourth decade of life	Second to third decade of life	≥20 years	<40 years
Family history of diabetes	Strong	Weak	Strong	Weak
History of childhood undernutrition	No	No	Yes	Yes
History of persistent undernutrition in adulthood	No	No	No	Yes
Socioeconomic status	High or middle	Low	High, middle or low	Low
BMI at presentation	≥25 kg/m^2^	<25 kg/m^2^	<25 kg/m^2^	<18.5 kg/m^2^
Ketonuria or DKA at onset	Present and unprovoked	Absent	Present with trigger factors	Absent
Pancreatic beta cell secretory function	Reduced with improvement after DKA resolution	Low (between T1D and T2D)	Low (between T1D and classical T2D, but greater than in MRD and FCPD)	Very low (lower than in T2D-NOV but greater than in T1D)
Pancreatic morphology on imaging	Normal (no calcifications, ductal dilatation or fibrosis)	Intra-pancreatic calcifications with large ductal dilatation	Normal (no calcifications, ductal dilatation or fibrosis)	Normal (no calcifications, ductal dilatation or fibrosis)
Peripheral insulin sensitivity	Low	High	High	High
Visceral and total body adiposity on imaging or BIA	Increased	Low	Low	Very low

BIA, bioimpedance analysis; DKA, diabetic ketoacidosis; T1D, type 1 diabetes; T2D, type 2 diabetes; T2D-NOW, type 2 diabetes in individuals without overweight or obesity

The review describes the epidemiology, clinical characteristics, aetiopathogenesis and management of the four atypical diabetes subtypes. It also addresses the challenges associated with precision diagnosis and management of these atypical diabetes subtypes within the African context and proposes region-specific pragmatic solutions. It also acknowledges that robust longitudinal research is needed to determine the incidence and prevalence of these diabetes subtypes and inform appropriate management guidelines for African populations. Ultimately, this review aims to enhance healthcare professionals’ understanding of these atypical diabetes subtypes in Black African populations, to support early, accurate diagnosis and the implementation of tailored treatment strategies. The terms ‘African’, ‘Black African’ and ‘African ancestry’ in the context of this review are used as operational definitions to synthesise the literature rather than to imply a homogeneous group of individuals with similar underlying biology and lived experiences.

## KPD in Black African populations

### KPD is more common in populations of African ancestry

KPD has been widely described in Black African populations, particularly among West African individuals [3–10]. It is also observed in other populations of African ancestry, such as African American and African Caribbean populations, as well as Hispanic populations, White populations of European origin and Asian populations [11–13]. KPD has previously been referred to as type 1.5 diabetes and Flatbush diabetes [14].

### KPD is considered a hybrid form of diabetes by the WHO

According to the 2019 WHO classification of diabetes, KPD is classed as a hybrid form of diabetes [15]. It is a heterogeneous condition comprising two major subgroups based on islet autoantibody status and beta cell secretory reserve at presentation. These are A–β+ KPD (A–: negative for islet cell antibodies; β+: preserved beta cell function) and A–β– KPD (A–: negative for islet cell antibodies; β–: reduced beta cell function) KPD [16].

### In clinical practice, diagnosing KPD is challenging and its misclassification is common

KPD typically presents with severe hyperglycaemia and transient, index episodes of unprovoked diabetic ketoacidosis (DKA) in the absence of islet cell autoimmunity [17, 18]. In general, individuals presenting with KPD are typically male in their fourth decade of life or younger and report a strong family history of diabetes [19] (Table 1). Women with KPD often have ovarian dysfunction, suggesting a protective role of oestrogen against oxidative stress-induced beta cell injury [13, 20].

At presentation, individuals with KPD often exhibit an overweight or obese phenotype similar to that of type 2 diabetes, with severe hyperglycaemia requiring insulin therapy for 1–3 months [17, 18]. Many achieve insulin independence and remission following resolution of DKA and thereafter may be managed with dietary interventions or oral glucose-lowering agents [17, 18]. From a clinical perspective, the presence of an unprovoked index episode of DKA and early attainment of diabetes remission following DKA resolution may help differentiate KPD from classical type 2 diabetes [21].

Metabolically, KPD is marked by reduced basal and stimulated insulin secretion and hyperglucagonaemia, indicating both alpha cell and beta cell dysfunction [4, 7]. However, with resolution of the acute episode of DKA, beta cell function often normalises [17].

In a small, selectively sampled study of Black African adults from Cameroon with different diabetes subtypes, no differences in beta cell function were observed between individuals with KPD and those with classical type 2 diabetes. These findings reveal that it may be difficult to distinguish KPD from classical type 2 diabetes based on beta cell functional status in individuals with long-standing diabetes [22]. Normal basal and stimulated C-peptide concentrations and clinical features such as acanthosis nigricans are predictive of future long-term insulin independence in individuals with KPD [10, 23, 24].

Despite the presence of reduced beta cell function in individuals with KPD, multi-organ insulin resistance is also common at the time of presentation and remission of an acute hyperglycaemic episode [5, 10].

Genetic studies of individuals with KPD demonstrate a low frequency of type 1 diabetes-associated HLA susceptibility alleles and a lower type 1 diabetes genetic risk score [3, 25]. Therefore, in the presence of DKA, in addition to islet cell antibody testing, genetic risk scores may be useful in differentiating type 1 diabetes from KPD.

### Aetiopathogenesis of KPD

#### Increased oxidative stress and genetic polymorphisms play an important role

The pathophysiology of KPD involves multiple putative mechanisms. Increased oxidative stress in pancreatic beta cells due to functional glucose-6-phosphate dehydrogenase (G6PD) deficiency, with a subsequent reduction in beta cell insulin secretion, has been implicated in the aetiopathogenesis of KPD [9]. In a study by Sobngwi et al that investigated G6PD erythrocyte enzyme activity and beta cell secretory function in a cohort of male participants of West African ancestry with KPD (*n*=59), type 2 diabetes (*n*=59) or normoglycaemia (*n*=55), a higher prevalence of G6PD deficiency was observed in participants with KPD (42.3%) than in those with type 2 diabetes (16.9%) and normoglycaemia (16.4%) [9]. G6PD catalyses the first step of the pentose phosphate pathway, which is important in the production of NADPH, a potent intracellular antioxidant that protects cells from oxidative stress [26].

Polymorphisms in genes essential in pancreatic beta cell development, differentiation and regeneration, such as *PAX4* (Arg133Trp) (encoding PAX4) and *NEUROG3* (encoding neurogenin 3), also play a role in the aetiopathogenesis of KPD in African populations [20, 27]. PAX4 is a transcription factor that plays a critical role in the differentiation of embryonic pancreatic progenitors into insulin-producing beta cells [27]. Neurogenin 3 is a transcription factor that is expressed in endocrine progenitor cells and controls pancreatic beta cell differentiation and regeneration [28]. As a result, *PAX4* and *NEUROG3* genetic polymorphisms, which are highly prevalent in West African and African American populations, are associated with marked beta cell secretory dysfunction and increased episodes of unprovoked DKA in individuals with KPD [27, 28].

#### Role of human herpesvirus type 8 and arginine deficiency in the pathogenesis of KPD

Human herpesvirus type 8 (HHV-8), a highly endemic virus in Africa [29], has also been shown to play a role in the aetiopathogenesis of KPD in African populations. In a study by Sobngwi et al, antibodies against latent and lytic HHV-8 antigens and HHV-8 viraemia were assessed in 81 individuals with KPD, 106 individuals with non-ketotic type 2 diabetes and 90 healthy control participants (all matched for age, sex and African region of origin). HHV-8 antibodies were found in 71 individuals (87.7%) with KPD compared with 16 individuals (15.1%) with non-ketotic type 2 diabetes and 36 control participants (40.0%). At initial presentation with DKA, HHV-8 genomic DNA was identified within peripheral blood mononuclear cells in six of 13 individuals (46.2%) with KPD and in no individual with non-ketotic type 2 diabetes. Additionally, viral proteins were found in human pancreatic beta cells that were cultured for 4 days in the presence of HHV-8 [8]. HHV-8 infection in individuals with KPD is associated with pancreatic beta cell dysfunction through the induction of endoplasmic reticulum and oxidative stress in beta cells, and through direct viral-related beta cell damage [8, 30].

Additionally, low arginine bioavailability during episodes of acute hyperglycaemia has been suggested to explain the onset of KPD in some individuals [31]. Arginine deficiency develops as a result of increased consumption through hydrolysis (to form ornithine), reduced dietary intake or upregulation of gut arginase activity [32]. Arginine is a semi-essential amino acid that potentiates glucose-stimulated insulin secretion by pancreatic beta cells [33].

### Management of KPD

Acute management of KPD is similar to that of DKA and is based on established treatment guidelines and protocols [17, 18]. Following resolution of DKA and discharge from hospital, insulin therapy (preferably a basal–bolus insulin regimen) should be maintained for at least 10 weeks regardless of pancreatic beta cell function to avoid DKA recurrence [17, 18].

Where possible, assessment of beta cell function by measurement of fasting and/or stimulated C-peptide concentrations should be performed 1–3 weeks after resolution of DKA to guide the subclassification of KPD (A–β+ KPD and A–β– KPD subtypes). Normal fasting and stimulated C-peptide of >0.33 nmol/l and >0.50 nmol/l, respectively, signify normal pancreatic beta cell function (as seen in individuals with the A–β+ KPD subtype) and predict safe insulin withdrawal and future insulin independence [17, 18].

For long-term management of KPD, individuals should be counselled about healthy lifestyle modification. Those with A–β+ KPD can be safely switched from insulin to oral glucose-lowering therapy, preferably metformin monotherapy or metformin in combination with low-dose sulfonylureas or dipeptidyl peptidase 4 (DPP-4) inhibitors if optimal glycaemic targets are not achieved with metformin monotherapy. Individuals with A–β– KPD should be maintained on long-term insulin therapy [17, 18]. For both subtypes, long-term use of oral glucose-lowering therapies prolongs the duration of diabetes remission for 24–40 months and prevents future episodes of ketosis [18].

The rates of DKA relapse range from 9% in the first year to 90% within 10 years in individuals with non-insulin-dependent KPD, with approximately 50% becoming insulin dependent within 10 years [19]. The significant clinical risk factors for DKA relapse in these individuals are the presence of progressive hyperglycaemia and an inadequate increase in the beta cell secretory reserve [19, 34].

## FCPD in Black African populations

### FCPD is common in the tropical regions of developing countries

FCPD is an atypical diabetes subtype that has been reported in Black African populations [35–37]. There are limited epidemiological studies in Africa to document the true prevalence of FCPD and its trend over time. Most of the information on FCPD in Africa comes from case series and reports [35–37]. This diabetes subtype has also been widely described in Asian populations [38, 39].

It is a secondary form of diabetes resulting from non-alcoholic calcific pancreatitis that mainly occurs in tropical low-income settings [40, 41]. The WHO classifies FCPD among the diseases of the exocrine pancreas or as type 3c diabetes [15].

### FCPD is often misdiagnosed as type 1 diabetes and mismanaged in clinical practice

Table 1 summarises the main features of FCPD. FCPD typically manifests in the second to third decade of life in individuals with normal or low weight, with a strong male predominance (~70%). Common clinical features include recurrent epigastric pain that radiates to the back, signs of undernutrition and features of pancreatic exocrine insufficiency, such as steatorrhea (this may be absent in the setting of a low-fat diet) [40, 41]. Non-invasive radiological imaging modalities, such as abdominal x-ray, ultrasonography, computed tomography (CT) and MRI, reveal characteristic intra-pancreatic calcifications and large ductal dilatation (mainly involving the main pancreatic duct) [40, 41]. In clinical settings without MRI, ultrasonography and CT imaging are preferred to abdominal x-rays in the diagnostic work-up of chronic pancreatitis because of better sensitivity and specificity (69% sensitivity and 97% specificity for ultrasonography; 75% sensitivity and 91% specificity for CT imaging) [42].

Diabetes usually develops later in the course of the condition due to progressive loss of pancreatic beta cell mass. Despite the presence of severe hyperglycaemia at presentation, people with FCPD are ketosis resistant, even with insulin withdrawal [40, 41]. This ketosis resistance is attributed to the partial preservation of pancreatic beta cells, concomitant pancreatic alpha cell damage with reduced glucagon concentrations, resistance to hepatic ketogenesis and subcutaneous adipose tissue lipolysis, reduced non-esterified fatty acid levels due to loss of or limited subcutaneous fat, and carnitine deficiency [40, 41].

### FCPD is associated with several pancreatic and extra-pancreatic complications

The common complications of FCPD include severe and difficult to control hyperglycaemia with marked glycaemic variability (brittle diabetes), microvascular diabetes complications such as diabetic retinopathy, nephropathy and neuropathy (including autonomic neuropathy), malnutrition with deficiency of fat-soluble vitamins (vitamins A, D, E and K), and pancreatic osteopathy (due to vitamin D deficiency in some or related to the degree of steatohorrea). Because of the low prevalence of cardiovascular risk factors such as hypertension and dyslipidaemia, macrovascular diabetes complications are rare [40, 41]. The prevalence of the above microvascular diabetes complications in individuals with FCPD is almost comparable to that seen in type 2 diabetes and other forms of chronic pancreatic diabetes [40, 41]. However, compared with other forms of chronic pancreatitis, a serious complication associated with FCPD is pancreatic adenocarcinoma. Prospective studies have reported a 100-fold increase in the risk of this condition in individuals with FCPD compared with control individuals [41]. Because of this increased risk, serial monitoring with CA19-9 testing and imaging (abdominal ultrasound or CT scans) is recommended [40, 41].

### Aetiopathogenesis of FCPD

#### Increased oxidative stress and genetic mutations play a central role

The aetiopathogenesis of FCPD remains poorly understood; however, several hypotheses have been proposed. People with FCPD have increased levels of oxidative stress and lipid peroxidation with relative deficiency of cellular antioxidants and micronutrients such as zinc [40, 43].

Familial clustering of FCPD suggests a potential role of genetic predisposition in its aetiopathogenesis. Recent studies have shown an association between FCPD and mutations in *SPINK1*, encoding the serum protease inhibitor Kazal type 1, *PRSS1* and *PRSS2*, which encode cationic trypsinogen and anionic trypsinogen, respectively, and *CTRC*, encoding chymotrypsinogen C [44–46].

Early studies conducted mainly in Kerala, India, proposed malnutrition and cassava (containing cyanogenic glycosides) consumption as possible underlying causes of FCPD [47]. However, recent observations and several studies have disproved these two hypotheses because FCPD has been observed in many regions of the world where cassava is not a staple food, and individuals with FCPD generally have defective detoxification of cyanogen whether they consume cassava or not [38, 48–50]. The observed malnutrition is regarded as a consequence of the condition, rather than its cause, due to low energy intake and malabsorption [38, 48–50].

### Management of FCPD

Broadly, the treatment of FCPD focuses on achieving optimal glycaemic management, correcting macro- and micronutrient deficiencies and addressing pancreatic exocrine insufficiency [41].

People with FCPD experience marked glycaemic variability with significant postprandial glucose excursions [51]. Basal and stimulated glucagon concentrations are low in individuals with FCPD because of the concomitant pancreatic alpha cell damage [52]. However, some studies have reported a paradoxical elevation in basal and stimulated glucagon concentrations in individuals with FCPD [53], particularly in the subset of those with early disease. In the presence of an atrophic pancreas, it has been hypothesised that the gut is the source of this extra-pancreatic hyperglucagonaemia, following activation of the proglucagon gene in the gut [54].

Because of the clinical factors above, most people with FCPD will require insulin therapy for glycaemic management. If available and affordable, insulin analogues administered using a basal–bolus approach (with regular self-monitoring of blood glucose) are preferred because of their better side-effect profiles (fewer episodes of hypoglycaemia and glycaemic variability). Oral glucose-lowering agents, preferably low-dose sulfonylureas with a low risk of hypoglycaemia such as gliclazide, can be used as alternatives in some people with FCPD [41].

Although it is used occasionally in individuals with FCPD and overweight, metformin should generally be avoided in the treatment of FCPD because of its associated adverse effects, such as abdominal pain, diarrhoea and exacerbation of weight loss and vitamin B_12_ deficiency. Incretin-based therapies, such as DPP-4 inhibitors and glucagon-like peptide-1 receptor agonists, should be avoided because of the potential risk of pancreatitis [41].

Screening and management of deficiencies in micronutrients, such as vitamins A, D, K and B_12_, folate, zinc and iron, and a high-protein diet are recommended for individuals with FCPD [41]. Regarding the management of pancreatic exocrine insufficiency, pancreatic enzyme supplementation (500–1000 U/kg of lipase with each meal) is recommended. Proton pump inhibitors can be added if there is a poor response to pancreatic enzyme supplementation [41].

Abdominal pain can be managed using non-opioid or opioid analgesics and anti-spasmodic agents. In cases of intractable abdominal pain despite adequate analgesia and anti-spasmodic treatment, sphincterotomy or drainage procedures such as lateral pancreaticojejunostomy (Puestow procedure) can be performed [41].

## Type 2 diabetes in Black Africans without overweight or obesity

Type 2 diabetes in individuals without overweight or obesity (BMI of <25 kg/m^2^ or <23 kg/m^2^ in some African populations) is an atypical diabetes subtype commonly seen in Black African populations [55–57].

Currently, type 2 diabetes in adults is mainly considered a condition of middle and older age, associated with obesity and insulin resistance, following secular trends in BMI. However, in most LMICs, type 2 diabetes is often described in relatively young individuals with normal BMI [58, 59] (Table 1). The proportion of individuals with type 2 diabetes and a normal BMI varies with ethnicity, geographical location, level of socioeconomic development and BMI cut-offs used, with higher frequencies noted in Black African and Asian populations (24–32%) than in White populations in high-income countries (<8%) [59–61].

### Individuals with type 2 diabetes without overweight or obesity have lower markers of adiposity, the metabolic syndrome and beta cell function

Individuals with type 2 diabetes without overweight or obesity have phenotypic characteristics that differ from those of individuals with overweight or obesity who develop type 2 diabetes. In a study of adult Ugandans with new-onset diabetes, those individuals with a BMI <25 kg/m^2^ were more likely than individuals with a BMI ≥25 kg/m^2^ to be male and have higher HbA_1c_ levels and lower levels of markers of the metabolic syndrome (hypertension, hyperuricaemia and hyperleptinaemia), adiposity (waist:hip ratio and visceral adiposity) and beta cell secretory function (oral insulinogenic index and 2 h post-glucose load serum C-peptide concentrations) [57].

### Aetiopathogenesis of type 2 diabetes in individuals without overweight or obesity

#### Early-life exposures and epigenetic changes play a crucial role

Several hypotheses have been proposed to explain the aetiopathogenesis of type 2 diabetes in individuals without overweight or obesity. It is thought that type 2 diabetes in these individuals develops due to a complex interplay between early-life environmental exposures, epigenetics and genetics. As described by the developmental origins of health and disease (DOHaD) hypothesis, early-life exposures (peri-conception, in utero, infancy and childhood), such as maternal and childhood undernutrition, chronic infections and excess maternal stress, influence lifelong health and capacity through permanent effects on the growth, structure and function of most key body organs including the pancreas and kidney (early-life programming) [62, 63].

Emerging evidence shows a strong link between a history of early-life and persistent undernutrition and the onset of diabetes [64–66]. These exposures induce epigenetic changes such as DNA methylation and histone modifications, which are inherited across generations. The epigenetic changes adversely affect the growth, differentiation and function of the endocrine pancreas [62, 63].

#### Genetic polymorphisms and the gut microbiome may also play an important role

An association between SNPs in or near the *CDKAL1*, *CDKN2BAS*, *KCNQ1*, *TCF7L2*, *CDC123*/*CAMK1D*, *HHEX*, *TCF2* and *KCNJ15* genes and type 2 diabetes in individuals without overweight or obesity has also been suggested in some studies [67, 68]. The *CDKAL1*, *CDKN2BAS*, *KCNJ15* and *KCNQ1* genes are actively expressed in pancreatic beta cells and play an integral role in pancreatic beta cell survival and secretory function [69].

Emerging evidence also suggests an influence of the gut microbiome on the aetiopathophysiology of type 2 diabetes in individuals without overweight or obesity [70]. In one study, reduced abundance of *Akkermansia muciniphila* was observed in non-overweight Chinese individuals with type 2 diabetes compared with their non-overweight counterparts without type 2 diabetes [71]. The abundance of *A. muciniphilia* correlates inversely with serum 3β‐chenodeoxycholic acid (βCDCA) levels and positively with insulin secretion [71, 72]. Importantly, βCDCA is associated with increased inhibition of insulin secretion [71].

#### Management of type 2 diabetes in individuals without overweight or obesity

##### Selection of glucose-lowering therapies should be individualised

Evidence on how to manage type 2 diabetes in African individuals without overweight or obesity is limited. Because of the predominance of features suggestive of pancreatic beta cell secretory dysfunction with minimal or no insulin resistance, insulin therapy (in individuals with confirmed insulin deficiency) or oral glucose-lowering agents that stimulate beta cell insulin secretion, such as newer generation sulfonylureas and DPP-4 inhibitors (preferably in individuals without insulin deficiency), may be used [73]. Additionally, studies conducted on White European and Asian populations with type 2 diabetes have demonstrated an optimal response to DPP-4 inhibitors and sulfonylureas in individuals with a non-obese phenotype [74, 75].

Metformin and glucagon-like peptide-1 receptor agonists should be avoided because both drugs worsen weight loss and sarcopenia [76]. The use of sodium–glucose cotransporter 2 inhibitors in individuals without overweight or obesity is associated with an increased risk of euglycaemic DKA, and these therapeutics should be used cautiously when indicated [77].

Robust clinical trials in Black African populations, known to have a high burden of non-obese or non-overweight type 2 diabetes, are urgently required to guide optimal care.

## MRD in Black African populations

### MRD is prevalent in African countries with food insecurity

MRD is another atypical diabetes subtype that has been described in studies and case reports from African countries with a history of prolonged periods of famine, such as Ethiopia, Uganda, Rwanda and Nigeria [78–84].

The WHO removed MRD from its classification of diabetes in 1999, citing a lack of compelling evidence that malnutrition causes diabetes [85]. However, current evidence shows an association between early-life undernutrition and the onset of diabetes [64–66].

With this growing evidence, an international consensus meeting was held in Vellore, India, in January 2025 to discuss atypical lean diabetes. During this 2 day meeting, a group of global diabetes experts unanimously agreed to recognise MRD as a distinct diabetes subtype with its own diagnostic criteria and treatment approach. During this consensus meeting, the term type 5 diabetes was proposed as an alternative name for MRD [86].

During the April 2025 IDF World Diabetes Congress in Bangkok, Thailand, the IDF officially recognised MRD as a distinct diabetes subtype that occurs in low-income settings. It also recognised the proposed alternative name for MRD of type 5 diabetes and launched the Type 5 Diabetes Working Group to develop formal diagnostic criteria and therapeutic guidelines for this atypical subtype [87].

### Individuals with MRD display phenotypic characteristics distinct from those of type 1 and type 2 diabetes

MRD is characterised by distinctive phenotypic features that do not align with the conventional description of classical type 1 and type 2 diabetes. These include a relatively early age at onset (<40 years) with a history of early-life undernutrition with persistent undernutrition in adulthood, low BMI of <18.5 kg/m^2^ or <19 kg/m^2^ in Asian populations, socioeconomic deprivation, absence of family history of diabetes, resistance to DKA despite withdrawal of insulin therapy, presence of a structurally normal pancreas (no calcifications, ductal dilatation, fibrosis or inflammation on imaging), a greater degree of beta cell dysfunction, and absence of insulin resistance, visceral adiposity and any known cause of an autoimmune or genetic form of diabetes [2, 78–80, 86, 88–90] (Table 1).

Recent evidence on MRD has re-ignited the debate about whether it is a distinct diabetes subtype deserving its own classification or an extreme form of type 2 diabetes, with a clinical overlap with type 2 diabetes in non-overweight or non-obese individuals [91–93]. To conclusively answer this clinical question, global diabetes registries and/or adequately powered longitudinal studies in countries where MRD is prevalent are required to fully understand the clinical characterisation, pathogenesis and evidence-based management of this diabetes subtype.

### Aetiopathogenesis of MRD

#### Prior and persistent undernutrition and antioxidant deficiency play a significant role

As posited by the DOHaD theory, early-life (in utero and childhood) and persistent adulthood undernutrition adversely affect the development and function of the pancreas [62, 63]. Undernutrition is also associated with antioxidant deficiency due to lower levels of vitamins A, E and C, as well as reduced catalase and glutathione peroxidase activity. This increases oxidative stress within the pancreatic beta cells, leading to an increase in apoptosis and a reduction in beta cell mass [86, 89]. Additionally, undernutrition-induced chronic oxidative stress is associated with dysfunction of two transcription factors, PDX-1 and MafA, resulting in defective insulin gene expression and pancreatic beta cell proliferation and function [86, 89].

### Management of MRD

#### Comprehensive nutritional rehabilitation and selection of appropriate glucose-lowering therapies are integral

There is a lack of evidence on how to manage MRD and further high-quality research is needed to guide healthcare professionals. Because severe pancreatic beta cell dysfunction is common in individuals with MRD [80, 88, 89], assessment of pancreatic beta cell function by fasting or random serum C-peptide measurement is helpful to guide the selection of appropriate glucose-lowering therapy.

For clinically stable individuals with normal serum C-peptide concentrations (fasting or random C-peptide concentrations of ≥0.25 nmol/l and ≥0.6 nmol/l, respectively), insulin secretagogues, such as sulfonylureas and DPP-4 inhibitors, may be effective glucose-lowering therapies. Insulin therapy, preferably using insulin analogues due to their associated low risk of hypoglycaemia, may be effective in individuals with MRD and low serum C-peptide concentrations (fasting or random C-peptide concentrations of <0.25 nmol/l and <0.6 nmol/l, respectively).

An individualised nutritional management plan that includes adequate protein, essential minerals and vitamin replacement is an important component in the management of individuals with MRD [86].

## Precision diagnosis and treatment of atypical diabetes subtypes in African populations: current gaps and proposed solutions

As diabetes is a highly heterogeneous condition, it is important to accurately diagnose, classify and manage the various subtypes appropriately to achieve optimal treatment outcomes. Clinical work-up of an individual with a suspected atypical diabetes subtype requires a comprehensive history, focused clinical examination (to identify any stigmata of atypical diabetes subtypes) and, where available, diagnostic tests such as islet autoantibody tests, a C-peptide test, a lipid profile panel, an oral glucose tolerance test or mixed-meal tolerance test and genetic tests [21].

Despite the high frequency of atypical diabetes subtypes in Africa [2], studies on the precision management of diabetes in Africa are lacking. Most essential diagnostic tests, such as islet autoantibody, C-peptide and genetic tests (especially for monogenic diabetes), are not available in most African countries. Access to advanced imaging modalities for better assessment of body adiposity and organ morphology, such as dual-energy x-ray absorptiometry (DEXA) scans and MRI, is also limited in most clinical settings in Africa. Furthermore, most countries in Africa have healthcare systems that are not adapted to chronic disease management, with an absence of universal health coverage and inequities in the distribution of diabetes diagnostic technologies and monitoring and treatment services, across both urban and rural settings [94]. These broader sociocultural, structural and contextual health system factors can collectively impact the diagnosis and classification of these different forms of diabetes and together deter the advancement of precision diabetes care in Africa.

To address these gaps, we propose practical solutions specific to the African region. Access to affordable clinical tests, such as C-peptide and islet autoantibody testing, and advanced imaging modalities should be improved to aid in the precise diagnosis of specific atypical diabetes subtypes. As Africa has the greatest genetic diversity globally, there is a need to set up local centres of excellence for genetic research and improve the capacity to perform genetic tests relevant to the African population. This will help to improve the precise diagnosis of genetic forms of atypical diabetes, as well as uncover previously unknown pathogenic pathways, to guide the development of novel precision therapies. Importantly, the expansion of such precision diagnostics and genetic research in Africa should be grounded in ethical and governance principles that prioritise African leadership and ownership, culturally responsive research practices and accountability to African populations as proposed in the ethics and governance framework for best practice in genomic research and biobanking in Africa developed by the H3Africa Consortium [95].

The approaches outlined above draw on available evidence from the published literature and international guidelines such as National Institute for Health and Care Excellence (NICE) [96] and ADA [97] recommendations, alongside the authors’ clinical and research experience in African and other resource-constrained settings. These are intended to address the key gaps in current diabetes care and research. The development of effective and sustainable strategies will require approaches that are shaped by local priorities and implemented through collaboration with people living with diabetes, healthcare professionals, researchers and policymakers.

Further multi-country collaborative research on the epidemiology, precision characterisation (phenotypic, genetic, epigenetic and metabolomic) and management of diabetes (including atypical diabetes subtypes) in Black African populations is urgently needed to inform practice and policy in the region. These studies must adopt a more standardised and context-sensitive approach to defining ethnicity in African populations, given the vast heterogeneity that exists across the continent.

## Conclusion

Diabetes phenotypes differ globally with atypical forms increasingly reported in Black African and Asian populations. In the Black African population specifically, KPD, FCPD, type 2 diabetes in non-overweight or non-obese individuals, and MRD have been described. These diabetes subtypes exhibit distinct phenotypic characteristics. To enhance the precision diagnosis and management of these subtypes in Africa, improved access to essential clinical, genetic and advanced imaging tests is required. Furthermore, greater collaborative research aimed at robustly characterising and developing affordable precision approaches to manage these atypical diabetes subtypes is warranted.

## Data Availability

No dataset is available for this review article. The studies reviewed are published and available online.

## Abbreviations

DKA: Diabetic ketoacidosis
DOHaD: Developmental origins of health and disease
DPP-4: Dipeptidyl peptidase 4
FCPD: Fibrocalculous pancreatic diabetes
G6PD: Glucose-6-phosphate dehydrogenase
HHV-8: Human herpesvirus type 8
KPD: Ketosis-prone diabetes
LMICs: Low- and middle-income countries
MRD: Malnutrition-related diabetes
